# KLF6 facilitates differentiation of odontoblasts through modulating the expression of P21 in vitro

**DOI:** 10.1038/s41368-022-00172-6

**Published:** 2022-04-14

**Authors:** Zhuo Chen, Wenzhi Wu, Chen Zheng, Yanhua Lan, Huizhi Xie, Zhijian Xie

**Affiliations:** Stomatology Hospital, School of Stomatology, Zhejiang University School of Medicine, Clinical Research Center for Oral Diseases of Zhejiang Province, Key Laboratory of Oral Biomedical Research of Zhejiang Province, Cancer Center of Zhejiang University, Hangzhou, China

**Keywords:** Differentiation, Cell proliferation

## Abstract

Multiple signaling pathways are involved in the regulation of cell proliferation and differentiation in odontogenesis and dental tissue renewal, but the details of these mechanisms remain unknown. Here, we investigated the expression patterns of a transcription factor, Krüppel-like factor 6 (KLF6), during the development of murine tooth germ and its function in odontoblastic differentiation. KLF6 was almost ubiquitously expressed in odontoblasts at various stages, and it was co-expressed with P21 (to varying degrees) in mouse dental germ. To determine the function of *Klf6*, overexpression and knockdown experiments were performed in a mouse dental papilla cell line (iMDP-3). *Klf6* functioned as a promoter of odontoblastic differentiation and inhibited the proliferation and cell cycle progression of iMDP-3 through *p21* upregulation. Dual-luciferase reporter assay and chromatin immunoprecipitation showed that *Klf6* directly activates *p21* transcription. Additionally, the in vivo study showed that KLF6 and P21 were also co-expressed in odontoblasts around the reparative dentin. In conclusion, *Klf6* regulates the transcriptional activity of *p21*, thus promoting the cell proliferation to odontoblastic differentiation transition in vitro. This study provides a theoretical basis for odontoblast differentiation and the formation of reparative dentine regeneration.

## Introduction

Tooth development is impelled by the epithelial-mesenchymal interaction, and it includes initiation, bud, cap, and bell stages.^1^ During this process, adjacent mesenchymal cells are induced by signals from the basement membrane of the epithelium to differentiate into odontoblasts.^2^ Odontoblasts are essential for producing non-collagenous proteins, such as dentin matrix protein 1 (DMP1) and dentin sialophosphoprotein (DSPP). They are also positive participants in dental tissue mineralization.^3,4^ In mature teeth, mesenchymal stem cells in the dental pulp will differentiate into odontoblasts and secrete mineralized matrix under pathologic conditions. Multiple signaling pathways are hypothesized to have important functions in odontogenesis and dental tissue renewal.^5^ However, the details of these mechanisms remain unknown.

Krüppel-like factor (KLF) proteins comprise a zinc finger transcription factor family containing highly conservative domains with three Cys2–His2 zinc finger.^6^ Previous studies have reported that KLF4, KLF5, and KLF10 were involved in odontoblastic differentiation.^7–10^ KLF4 is the most thoroughly investigated member. Lin et al. proposed that KLF4 promotes odontoblastic differentiation by specifically combining with the CACCC elements on the DMP1 promoter and transactivating its expression.^11^ A recent study reported that mutant molars of *Wnt1*-cre-mediated KLF4 conditional knockout mice exhibited significantly decreased thickness, density, and mineral deposition rate of dentin, which confirmed KLF4 is responsible for the mineralization and ultrastructure of dentin.^12^ However, functions of other KLFs in tooth development and odontoblastic differentiation are not yet clear.

KLF6 was first isolated from placental cells, which is encoded by four exomes on chromosome 10p15.^13,14^ KLF6 has a wide expression profile and regulates multiple biological behaviors, such as cell cycle arrest,^15,16^ adipogenesis,^17,18^ cell metabolism,^19^ and apoptosis.^16,20,21^ In general, KLF6 is considered a tumor suppressor gene.^14,22–25^ Moreover, *Klf6* is early expressed in mouse embryonic development, and the targeted knockout of *Klf6* induces impairment of embryonic stem cell differentiation into hepatocytes, angiogenesis, and vascularization failure.^26^ Thus, the *Klf6* gene plays a significant role in the development of organs arising from the endoderm. The expression pattern and function of KLF6 during odontogenesis remain unclear, however. Here, we hypothesized that KLF6 may participate in odontogenesis.

As a transcription factor, KLF6 forms a complicated transcriptional regulatory network. Further exploration of the underlying mechanisms will aid in understanding odontogenesis and how the protective response of dental pulp takes place under pathologic conditions. Therefore, this study aimed to investigate KLF6’s expression profile in mouse tooth germ and further clarify its association with odontoblastic differentiation.

## Results

### Expression profile of KLF6 and P21 during murine tooth development

Hematoxylin-eosin (H&E) staining was employed to show the morphology of tooth germs from E12.5 to PN5. Immunostaining revealed that KLF6 and P21 were expressed in various cell types during tooth germ development.

**Fig. 1:**
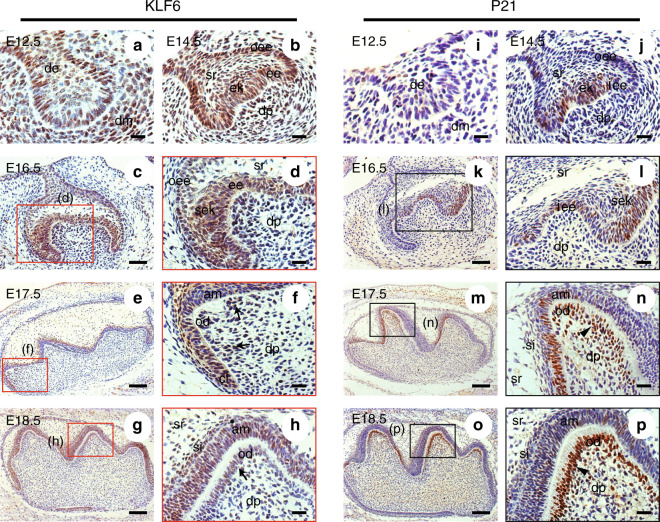
Immunohistochemistry of KLF6 and P21 in murine tooth germs in embryonic development. **a**, **i** At E12.5, the expressions of KLF6 and P21 were observed in the epithelium and mesenchyme. **b**, **j** At E14.5, KLF6 and P21 were expressed in the enamel epithelial cells, stratum reticulum, primary enamel knot, and dental papillae. **c**, **k** At E16.5, P21 and KLF6 were both expressed in the inner epithelium and secondary enamel knots (scale bar = 50 μm). **e**, **m** At E17.5, intense staining for KLF6 and P21 was observed in the polarizing ameloblasts of the inner enamel epithelium and preodontoblasts beneath the inner enamel epithelium of the cusp (scale bar = 100 μm). **g**, **o** At E18.5, KLF6 and P21 were strongly detected in the nuclei of the differentiating ameloblasts and odontoblasts (scale bar = 100 μm). **d**, **f**, **h** Images showing details of the red-framed areas in (**c**), (**e**), and (**g**), respectively. **l**, **n**, **p** Images showing details of black-framed areas in (**k**), (**m**), and (**o**), respectively. **a**, **b**, **d**, **f**, **h**, **i**, **j**, **l**, **n**, **p** Scale bar = 20 μm. OEE outer enamel epithelium, IEE inner enamel epithelium, DE dental epithelium, DM dental mesenchyme, DP dental papillae, EK enamel knot, SR stratum reticulum, SI stratum intermedium, CL cervical loop, SEK secondary enamel knot, AM ameloblast, OD odontoblast, Arrows, positive KLF6 expression in odontoblasts at different differentiation phases; Arrowheads, positive P21 expression in odontoblasts at different differentiation phases

At E12.5 (the bud stage), KLF6 and P21 were both positive in the epithelium and mesenchyme (Fig. 1a, i). At E14.5 (the cap stage), strong expressions of P21 and KLF6 were simultaneously found in the outer and inner enamel epithelium (Fig. 1b, j). They were also found in the primary enamel knot. The expression of KLF6 still exhibited a broad spectrum at this time; however, P21 was not detected in the stratum reticulum, dental follicle, and dental papillae (Fig. 1j).

At E16.5 (the early bell stage), KLF6 and P21 were mainly found in the inner epithelium and secondary enamel knots (Fig. 1d, l). KLF6 was expressed in the outer enamel epithelium, stellate reticulum, and mesenchymal cells beneath the inner enamel epithelium (Fig. 1d). At E17.5, in addition to the inner and outer enamel epithelial cells and the stratum reticulum, KLF6 and P21 were also predominantly expressed in the dental papilla (Fig. 1e, l).

At E18.5 (the late bell stage), KLF6 was observed in ameloblasts and odontoblasts of different differentiation stages, while the stratum intermedium and stratum reticulum both exhibited moderate KLF6 staining (Fig. 1h). At this stage, intense P21 staining was observed mainly within the nuclei of the ameloblasts and odontoblasts of the future cusp (Fig. 1p).

**Fig. 2:**
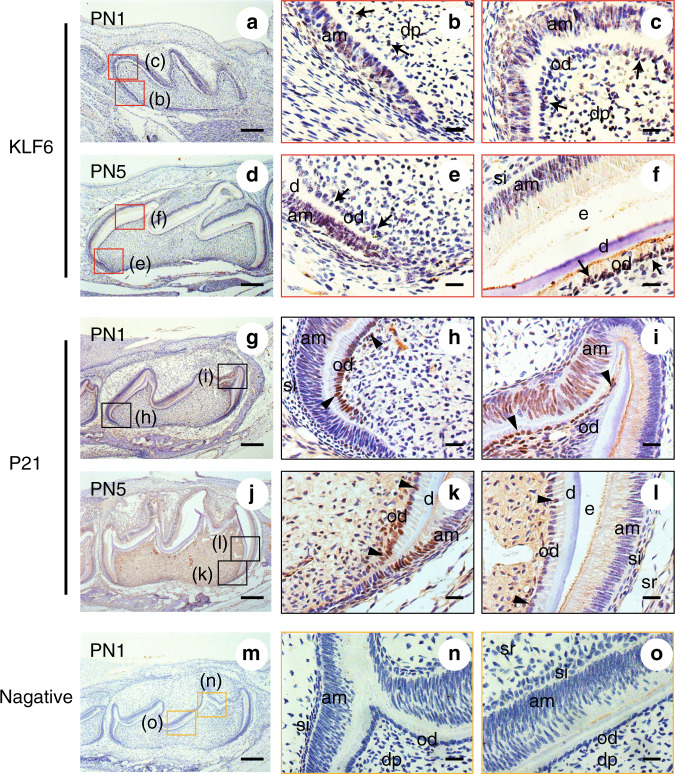
Immunohistochemistry of KLF6 and P21 in murine tooth germs during postnatal development. **a** At PN1, KLF6 was positively expressed in the preameloblasts/preodontoblasts, and in the polarizing, secretory, mature ameloblasts. **g** At PN1, P21 was expressed in the preameloblasts/preodontoblasts, polarizing and secretory odontoblasts/ameloblasts, and mature odontoblasts; it was absent, however, from the mature ameloblasts. **d**, **j** At PN5, KLF6 and P21 expression profile was similar to that at PN1. **m** A negative control was prepared by staining with IgG, and no positive immunostaining was detected. Scale bar = 200 μm. **b**, **c** Images showing details of the red-framed areas in (**a**). **e**, **f** Images showing details of red-framed areas in (**d**). **h**, **i** Images showing details of black-framed areas in (**g**). **k**, **l** Images showing details of black-framed areas in (**j**). **n**, **o** Images showing details of yellow-framed areas in (**m**). Scale bar = 50 μm. Arrows, positive KLF6 expression in odontoblasts at different differentiation phases. Arrowheads, positive P21 expression in odontoblasts at different differentiation phases

At PN1, KLF6 was positively expressed in the ameloblasts and odontoblasts of different differentiation stages (including the preameloblasts/preodontoblasts, polarizing, secretory, and mature ameloblasts/odontoblasts) (Fig. 2b, c). P21 was also expressed in the ameloblasts and odontoblasts of different differentiation stages; its staining was slight, however, in the mature ameloblasts (Fig. 2h, i). At PN5, the KLF6 and P21 expression patterns were similar to those at PN1 (Fig. 2e, f). Notably, P21 and KLF6 expressions were both strongly detected in the polarizing and secretory odontoblasts.

Furthermore, we also detected KLF6 and P21 expressions using immunofluorescence methods in mouse dental papilla cells at PN5. KLF6 and P21 were both expressed in odontoblasts of different differentiation stages and dental papillae (Fig. S1). Detection of KLF6 and P21 by immunofluorescence assays was consistent with the immunochemistry results (Fig. 2d–f, j–l).

### KLF6 and P21 expression patterns during odontoblastic differentiation in vitro

iMDP-3 cells were often used in studying the mechanism of odontoblast differentiation.^8,27^ To identify the odontogenic capability of iMDP-3 cells, specific markers related to odontoblastic differentiation, such as alkaline phosphatase (ALP), collagen I (COL1), osteopontin (OPN), osterix (OSX), and DMP1, were detected by immunofluorescence staining 14 days after odontogenic culture (Fig. S2a–o). ALP and alizarin red staining (ARS) revealed similar odontoblastic differentiation capacity of the cells with primary cells (Fig. S2p–s). These results are consistent with those of the previous study.^28^

**Fig. 3:**
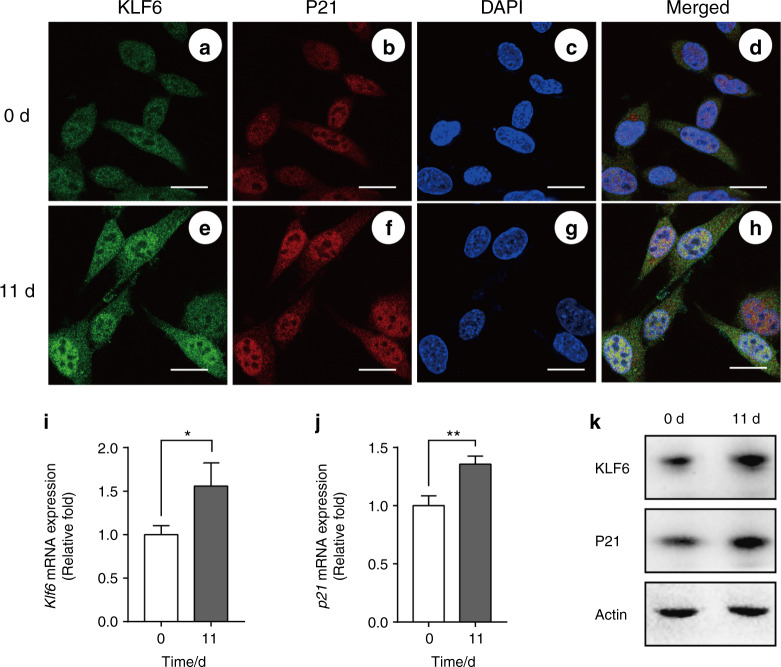
Expression patterns of KLF6 and P21 in iMDPC-3 cells. Co-expression of KLF6 and P21 in iMDP-3 cells after culturing the cells in DM for 11 days (**e**–**h**) and 0 days (**a**–**d**). **c**, **g** Nuclei of iMDPC-3 cells with DAPI. **d**, **h** Merged images. **i**–**k** mRNA and protein expression levels of KLF6 and P21. Bars = 20 μm. **P < 0.01, * *P* < 0.05, vs 0 day

Because KLF6 and P21 appeared to be co-expressed (to a degree) in tooth germ during murine tooth development, their co-expression in iMDP-3 cells during odontoblastic differentiation was investigated using immunofluorescence histochemical double staining. In the control group, both KLF6 (Fig. 3a, e) and P21 (Fig. 3b, f) were observed to be expressed in the nucleus and cytoplasm. After being cultured in a differentiation medium (DM) for 11 days, the cells had all become long and had a spindle-shaped appearance. Fluorescence intensities of both KLF6 and P21 were enhanced after being cultured in DM for 11 days (Fig. 3a, e, b, f). RT-qPCR and western bolt results further confirmed that KLF6 and P21 were upregulated after being cultured in DM (Fig. 3i–k). Therefore, KLF6 and P21 may function in the differentiation process in iMDP-3.

### Effects of Klf6 overexpression on gene expression, proliferation, apoptosis, and cell cycle

**Fig. 4:**
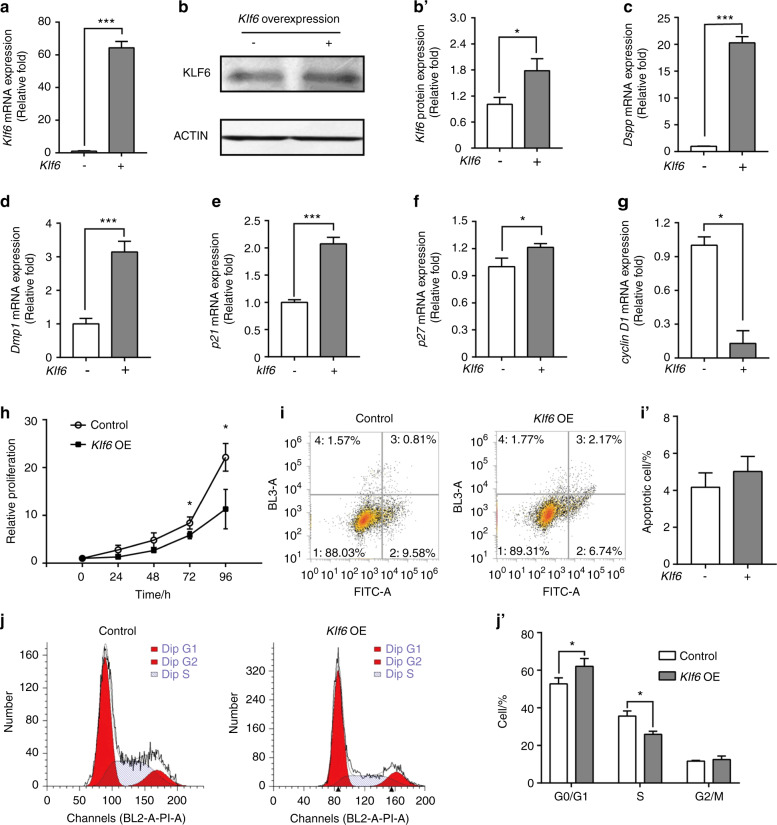
Effects of *Klf6* overexpression on gene expression in iMDP-3 cells. **a** mRNA levels of the *Klf6* after *Klf6* overexpression. **b**, **b**′ Protein expression levels of the *Klf6* gene. **c**–**g** mRNA expression levels of *Dspp, Dmp1, p21, p27*, and *cyclin D1*. **h** Cell proliferation was detected by a methyl-thiazol-tetrazolium assay in iMDP-3 cells 48 h after transfection. **i**, **i**′ The apoptotic rates of the two groups. **j**, **j**′ Cell cycle distribution. ***P < 0.001, **P < 0.01, *P < 0.05, vs control

Transfection assays were used to interrogate the regulatory effects of *Klf6* on iMDP-3 cells. iMDP-3 cells were transfected with either a *Klf6* overexpression plasmid (flag-hKLF6, Addgene) or the control plasmid (pCI-neo, Invitrogen). The overexpression group’s *Klf6* mRNA expression level was considerably higher than the control group’s 48 h after transfection (*P* < 0.001) (Fig. 4a). KLF6 protein level also increased, further confirming the efficiency of the *Klf6* overexpression (Fig. 4b, b’). Notably, our results showed that *Klf6* overexpression led to increased expressions of *Dspp* (*P* < 0.001) (Fig. 4c) and *Dmp1* (*P* < 0.01) (Fig. 4d).

We also investigated whether *Klf6* upregulation affects the expression of *p21* and several other cell cycle regulators, including *p27* and *cyclin D1*. We found that the expression of *p21* and *p27* were increased in the *Klf6* overexpression group (Fig. 4e, f), whereas *cyclin D1* expression was reduced (*P* < 0.05) (Fig. 4g).

Furthermore, using the methyl-thiazol-tetrazolium (MTT) assay, *Klf6* overexpression inhibited iMDP-3 cell proliferation at 72 and 96 h (Fig. 4h). Next, we conducted flow cytometry to analyze the function of *Klf6* in odontoblast apoptosis. The apoptotic rates of the *Klf6* overexpression and control groups were 4.17% ± 0.77% and 5.02% ± 0.815%, respectively (Fig. 4i, i’) (*P* > 0.05). This finding indicates that KLF6 has little effect on the apoptosis of iMDP-3 cells.

Moreover, the cell cycle was examined using flow cytometry (Fig. 4j). The control group had a lower average proportion of G0/G1 phase cells (52.77%  ±  2.25%) (*P* *<* 0.05)*.*  In the *Klf6* overexpression group, that proportion was 62.07%  ±  2.95%. Furthermore, 35.62%  ±  1.95% and 25.91%  ±  1.17% of the cells in the control and *Klf6* overexpression groups were in the S phase, respectively (*P*  < 0.05). The G2/M phase proportions were 11.63% ± 0.30% and 12.53% ± 1.29% for the control and *Klf6* overexpression groups, respectively (*P* > 0.05). These results suggest that *Klf6* overexpression increases the expression of *p21* and other cell cycle regulators in iMDP-3 cells, thereby resulting in cell proliferation inhibition and cell cycle block at the G1-S checkpoint.

### Effects of Klf6 knockdown on gene expression, proliferation, apoptosis, and cell cycle of iMDP-3 cells

**Fig. 5:**
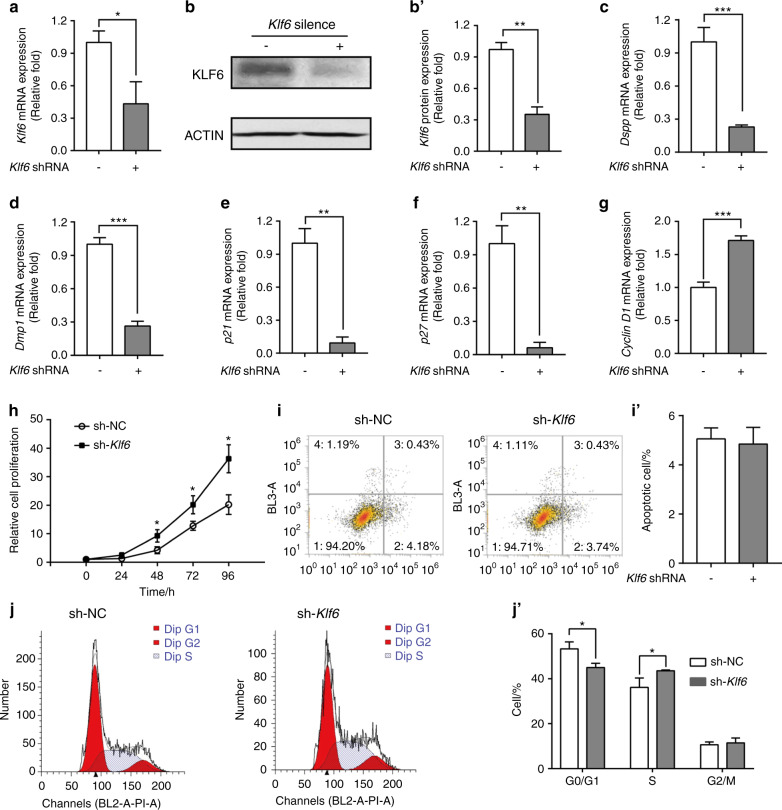
Effects of *Klf6* knockdown on gene expression in iMDP-3 cells. **a**–**b**′ mRNA and protein levels of *Klf6* after *Klf6* knockdown. **c**–**g** mRNA expression levels of *Dspp, Dmp1, p21, p27*, *cyclin D1*. **h** Cell proliferation was detected by a methyl-thiazol-tetrazolium assay in iMDP-3 cells 24 h after transfection. **i**, **i**′ The apoptotic rates of the two groups. **j**, **j**′ Cell cycle distribution examined by flow cytometry. ***P < 0.001, **P < 0.01, *P < 0.05, vs control

To identify whether *Klf6* upregulation is required for odontoblastic proliferation and differentiation, we transfected iMDP-3 cells with *Klf6* shRNA (sh-KLF6) or control shRNA (sh-NC). After 24 h of treatment with sh-KLF6, the *Klf6* mRNA expression level was decreased (*P* < 0.05) (Fig. 5a). This decreasing trend was further confirmed by western blotting (*P* < 0.01) (Fig. 5b, b’). Knockdown of *Klf6* exerted an opposite regulatory effect on the expressions of *Dspp*, *Dmp1*, *p21*, *p27*, and *cyclin D1* (Fig. 5c–g).

The MTT assay revealed that knockdown of *Klf6* promoted cell proliferation in iMDP-3 cells (Fig. 5h). The apoptotic rates of the *Klf6* knockdown and control groups were 4.85% ± 0.68% and 5.06% ± 0.45%, respectively (Fig. 5i, i’). However, there was no statistical difference between the two groups (*P* > 0.05).

Flow cytometry was also used to identify the cell cycle. The findings indicated that silencing *Klf6* promoted the progression of the iMDP-3 cells at the G1-S checkpoint. The G0/G1 phase proportions were 44.99% ± 1.90% and 53.26% ± 3.14% in the *Klf6* knockdown and control groups, respectively (*P* < 0.05). Conversely, the S phase proportions were 43.55% ± 0.34% and 36.12% ± 4.18% in the *Klf6* silencing and control groups, respectively (*P* < 0.05) (Fig. 5j). Based upon the results of both overexpression and knockdown experiments, we suggest that *Klf6* modulates the expression levels of *p21* and other cell cycle regulators in iMDP-3 cells, indicating that preodontoblasts exit the cell cycle loop and start differentiation.

### Klf6 directly regulated the transcriptional activity of p21 in iMDP-3 cells

**Fig. 6:**
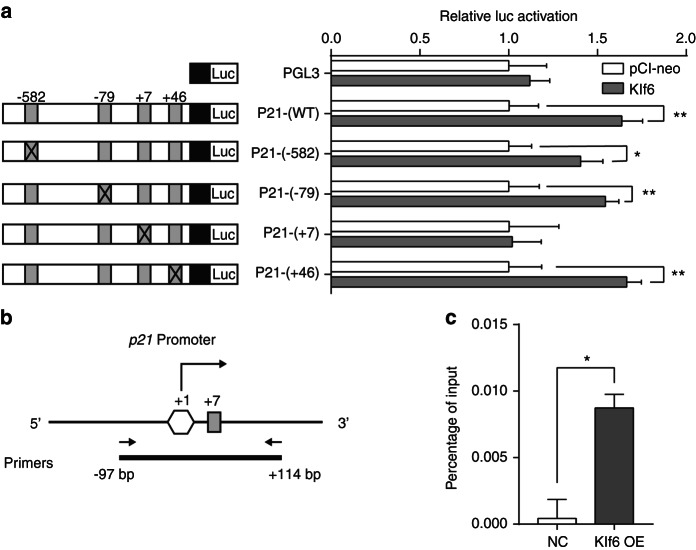
*Klf6* directly regulates the transcriptional activity of *p21*. **a** Dual-luciferase assays found that the wild-type plasmid, PGL3-*p21*-(WT), increases transcription 1.6-fold compared to the control. Moreover, mutant plasmids including PGL3-*p21*-(−582), PGL3-*p21*-(−79), and PGL3-*p21*-(+46), also upregulated the transcriptional activity. Only the cells transfected with PGL3-*p21*-(+7) and *Klf6* plasmid did not show a marked change of transcriptional activity. **b** The schematic diagram of the primers designed for ChIP-qPCR. **c** ChIP-qPCR verified that KLF6 directly combines with the promoter of p21 at +7 site in iMDP-3 cells. ***P < 0.001, **P < 0.01, *P < 0.05, vs control

Dual-luciferase and chromatin immunoprecipitation (ChIP) assays were performed to further identify whether *Klf6* directly regulates *p21* transcriptional activity and validate the main binding site on the *p21* promoter region. The cells transfected with PGL3-*p21*-(WT) and *Klf6* plasmid showed significantly stronger luciferase activity than the cells transfected with PGL3-*p21*-(WT) and pCI-neo plasmid (*P* < 0.01). This demonstrates that Klf6 can directly regulate the transcription of *p21* (Fig. 6). Bioinformatics analysis predicted that there are four possible *Klf6*-binding sites on the *p21* promoter region (Table. S2). To identify whether these sites are actually working, we mutated each site to construct four distinct recombinant *p21* reporter plasmids. The results revealed that only the cells transfected with PGL3-*p21*-(+7) and the *Klf6* plasmid did not show a substantial shift in transcriptional activity (*P* > 0.05). Thus, *Klf6* mainly activates *p21* transcription at the +7 site. Further, ChIP-qPCR was performed. We designed a pair of primers (from −97bp to +114 bp) covering the +7 site on the *p21* promoter (Fig. 6b). The ChIP-qPCR experiment showed that KLF6 protein was recruited to the *p21* promoter (Fig. 6c).

### KLF6 and P21 were also expressed in the odontoblasts around the reparative dentine

**Fig. 7:**
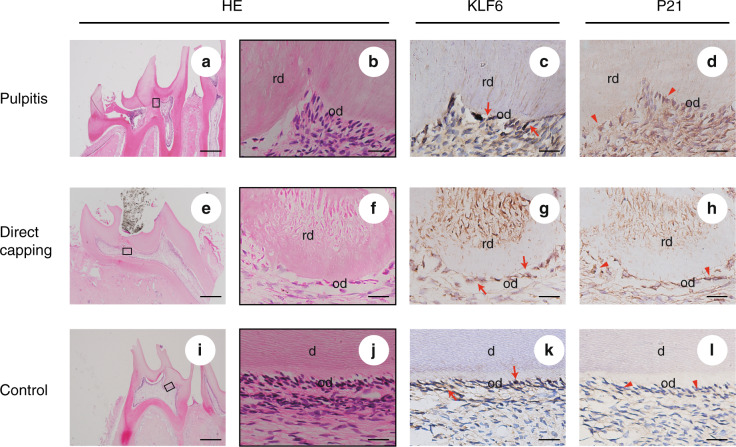
Expression patterns of KLF6 and P21 in rat pulp exposure models. **a** H&E staining of the pulpitis group. Necrotic pulp, substantial inflammatory cell infiltration, and reparative dentin were observed. **e** H&E staining of the direct capping group. Evident reparative dentin bridge formation was observed beneath the perforation site. **i** H&E staining of the control group showed normal pulp tissue. Bars = 500 μm. **c**, **g**, **k** Expression of KLF6 as seen using immunohistochemistry. **d**, **h**, **l** Expression of P21 using immunohistochemistry. **b**, **f**, **j** Images showing details of the black-framed areas in (**a**, **e**, and **i**), respectively. Bars = 25 μm. Arrows, positive KLF6 expression in odontoblasts; Arrowheads, positive P21 expression in odontoblasts; D dentin, RD reparative dentin, OD odontoblast

The above in vitro results show that *Klf6* directly activates *p21* transcription and regulates cell cycle and proliferation, thus allowing the iMDP-3 cells to enter the differentiation stage and gradually lose the proliferation ability. Consequently, this prompted us to become interested in the function of *Klf6* during reparative dentin formation in vivo. Therefore, we constructed a rat pulp exposure model to study in vivo KLF6 and P21 expression patterns. H&E staining was performed to identify the different histological characteristics. Necrotic pulp and substantial inflammatory cell infiltration were observed in the rat pulpitis model (Fig. 7a). Since chronic inflammation of the dental pulp was prolonged for 4weeks, reparative dentin was observed in the pulp chamber (Fig. 7b). In the direct capping group, reparative dentin bridge formation was observed beneath the perforation site (Fig. 7e, f). Immunohistochemical staining showed that KLF6 and P21 were co-expressed in odontoblasts of healthy pulp tissue (Fig. 7k, l). The KLF6 and P21 staining intensities were also strong in the odontoblasts around the reparative dentin in the direct capping and pulpitis groups (Fig. 7g, h). In addition, similar results were obtained by immunofluorescence double staining (Fig. S3). These results imply that both KLF6 and P21 may also participate in the process of rat reparative dentine formation.

## Discussion

During tooth development, odontoblasts can secrete mineralized matrix and mediate the formation of dentin. In carious teeth, dental pulp cells will differentiate into odontoblasts that can mineralize to form reparative dentin, thus inhibiting the progression of inflammation.^29^ Uncovering the mechanism of odontoblastic differentiation will help to open up new avenues for further development of treatments for dental diseases.

KLF6 has three C_2_H_2_ zinc fingers in the highly conserved C-terminal; it also recognizes similar sequences (GC-rich elements or the CACCC motif) of target genes.^14,16,18^ In our previous research, *Klf6* can promote odontoblastic differentiation partly through the activation of *Dspp* and *Dmp1*, which are both essential markers for odontoblastic differentiation and dentin mineralization.^27^ During dentinogenesis, preodontoblasts exist cell cycle, stop cell proliferation, and differentiate into odontoblasts.^30,31^ Therefore, proliferation and differentiation are inextricably associated with each other. To more fully explicate the complex *Klf6* regulatory network during odontoblastic differentiation, the role of *Klf6* in the switch from cell cycle exit to odontoblastic differentiation were examined. The results of this study demonstrate that *Klf6* regulates *p21* expression and functions as an inducer of odontoblastic differentiation in vitro by promoting iMDP-3 cells to exit the cell cycle loop and begin differentiation.

P21(Cdkn1a) is a kinase inhibitor protein that blocks the G1/S transition. It also is generally regarded as a downstream target of KLF6.^14,18^ We first investigated the co-expression profiles of KLF6 and P21 from E12.5 to PN5 and found that they are co-expressed to varying degrees in tooth germ during this process (Figs. 1,2). Previous studies have shown that P21 is predominantly expressed in the inner enamel epithelium and enamel knots.^32,33^ It is believed that P21 functions in blocking enamel knot cell proliferation.^33^ The P21 expression pattern in our study is consistent with these studies. Additionally, KLF6 and P21 were co-localized in the primary and secondary enamel knots. This highlights the possibility that KLF6 participates in regulating the enamel knot cell cycle by modulating P21 expression and is thus involved in cusp formation and tooth morphogenesis.

Moreover, a recent study indicated that P21 could be regarded as a marker of the early phases of odontoblast differentiation since the proliferating cells quit the cell cycle to differentiate into odontoblasts.^34^ Because both P21 and KLF6 were strongly expressed in the polarizing and secretory odontoblasts but weakly expressed in mature ameloblasts and odontoblasts at PN1 and PN5 (Fig. 2), KLF6 and P21 may participate in proliferation and differentiation of ameloblasts and odontoblasts.

As our in vivo study showed that KLF6 and P21 are co-expressed to varying degrees in mouse tooth germ, we further investigated their co-expression in iMDP-3 cells during odontoblastic differentiation. In our previous study, an increase in *Klf6* expression was detected during odontoblastic differentiation, reaching its highest level at 11 days.^27^ Thus, we selected the 11th day as the observational time point in the present in vitro experiment. After culturing iMDP-3 cells in DM for 11 days, the expressions of both KLF6 and P21 were higher compared with the control (Fig. 3). Thus, KLF6 and P21 may participate in the odontoblastic differentiation of iMDP-3 cells.

Additionally, gain- and loss-of-function assays were employed to validate the association between *Klf6* and *p21* and further investigate the mechanism through which *Klf6* regulates odontoblastic proliferation and differentiation. KLF6 plays an inhibitory role in cell proliferation in various normal and tumor cells.^25,35^ In hepatoma cell lines, KLF6 overexpression inhibits cell proliferation, migration, and angiogenesis.^36^ *Klf6* overexpression induced the inhibition of iMDP-3 cell proliferation and cell cycle arrest, whereas knocking down *Klf6* promoted cell proliferation and progression at G1-S transition (Figs. 4j’, 5j’). The cell apoptotic rates between the experimental and control groups showed no significant difference, however (Figs. 4i’, 5i’). Therefore, the function of *Klf6* in apoptosis regulation may not play a prominent role in iMDP-3 cell proliferation; rather, *Klf6* inhibits cell proliferation mainly via cell cycle arrest.

Moreover, forced expression of *Klf6* significantly induced *p21* and *p27* expression in iMDP-3 cells. Overexpression of KLF6 results in inhibition of rat lens epithelial cell proliferation by increasing P21 and P27 expression.^37^ Our results are in line with that previous study. Moreover, *p21* and *p27* are both important cell cycle inhibitors.^38^ Cell cycle progression is an essential part of cell proliferation. The G1-S checkpoint controls whether cells continue to the S phase or stop dividing and enter the G0 phase, leading to differentiation. Cyclin D1 is a crucial gene that regulates the G1-S transition.^39^ Complexes formed with Cyclin D1 and cyclin-dependent kinase 4 and 6 (CDK4 and CDK6) promote cell cycle progression by inhibiting the retinoblastoma protein.^40^ Furthermore, *cyclin D1* expression can be inhibited by *p21* and/or *p27* through transcriptional regulation.^38^ KLF6 also directly disrupts the interaction between Cyclin D1 and CDK4 and causes cell cycle arrest.^41^ We found that *Dspp* and *Dmp1* were notably upregulated by *Klf6* overexpression. *Dspp* and *Dmp1* are both essential markers for odontoblastic differentiation and dentin mineralization.^42^ The *Dspp* and *Dmp1* expression patterns we observed indicate that the iMDP-3 cells were in the process of differentiation. Therefore, we propose that *Klf6* regulates *p21* expression, accompanied by changes in the expression of other cell cycle genes (e.g., *p27* and *cyclin D1*), to promote the transition from proliferation to differentiation in iMDP-3 cells.

To clarify this mechanism, we performed dual-luciferase reporter assays and ChIP-qPCR in this study, and the results reveal that *Klf6* directly regulates the transcriptional activity of *p21* in iMDP-3 cells. Previous studies have shown that KLF6 directly transactivates the P21 promoter in an immortalized human embryonal fibroblast cell line (293 T) and that the binding sites for KLF6 have been reported to be located at −102 and −30 bp upstream from the P21 transcriptional initiation site in 293 T cells.^43,44^ Since the existence of tissue- and cell-specific regulatory sites have been well-proofed, our data show that the binding site at the +7 bp locus of *p21* promoter plays a prominent role in Klf6-mediated activation of *p21* (Fig. 6). Taken together, *Klf6* controls *p21* expression, thus inducing iMDP-3 to exit the cell cycle and differentiate into odontoblasts.

Finally, since odontoblasts are essential for reparative dentin formation, we further observed the expressions of KLF6 and P21 during reparative dentin formation in rat molars. Rats are the most frequently used animal for constructing pulp exposure animal models and studying potential dentinogenesis and mineralization mechanisms.^45,46^ KLF6 and P21 were expressed in the odontoblasts around the reparative dentin in vivo (Figs. 7 and S3). This suggests that KLF6 may also participate in reparative dentin formation. However, the detailed mechanism remains to be elucidated. These findings provide insight into the future possibility of using KLF6, an odontoblastic differentiation activator, to promote the formation of a reparative dentin layer and help to better understand the mechanism behind the transition from cell proliferation to odontoblastic differentiation.

Overall, *Klf6* is expressed in odontoblasts and ameloblasts at various stages of differentiation during murine tooth development and regulates odontoblast cell proliferation and differentiation in vitro. It also regulates the transcriptional activity of *p21*, thus promoting the transition from proliferation to differentiation in iMDP-3 cells. During reparative dentinogenesis, KLF6 was expressed in odontoblasts around the reparative dentin. The role of KLF6 as an activator of odontoblastic differentiation in vitro makes it a potential target for developing novel bioactive capping materials. Future studies should focus on investigating the specific KLF6 regulatory mechanism during pulp inflammation and reparative dentin formation.

## Materials and methods

### Preparation of tissue slices

All the procedures were approved by the Animal Ethics Committee of Zhejiang University (ZJU20200094). Adequate procedures were applied to reduce suffering and discomfort in the animals, and the studies were conducted in compliance with local laws and regulations. Kunming mice were obtained from Zhejiang University (Hangzhou, China). The day the vaginal plug was present was considered embryonic day 0.5 (E0.5), and the day of birth was considered postnatal day 0.5 (PN0.5). At each series development point before birth (from E12.5 to E18.5), three pregnant female mice were sacrificed and the heads of embryos were collected. Six young mice were sacrificed at each postnatal point (PN1 and PN5) and the mandibles of the mice were dissected. Each sample was promptly fixed in 4% paraformaldehyde buffer overnight. After decalcification by 10% ethylene diamine tetraacetic acid (EDTA), paraffin sections (4-μm) were prepared.

### Histological analysis and immunohistochemistry of tissue sections

Selecting at least six representative sections of each sample for follow-up experiments, and at least three samples per group were used. H&E staining was performed following routine protocols. Slices were deparaffinized with xylene before being rehydrated through gradient ethanol into water. Antigen was retrieved by LAB Solution (Polysciences) at 25 °C for 5 min. Immunohistochemistry was conducted using an UltraSensitive SP IHC kit (Maixin Biotechnology). Sections were pretreated with a peroxidase-blocking reagent for 10 min and incubated with goat serum to prevent nonspecific antibody binding. The sections were then incubated overnight with diluted KLF6 (1:50, Santa Cruz, # sc-7158) or P21 (1:200, Santa Cruz, # sc-817) mouse monoclonal antibodies at 4 °C. Negative controls were treated with mouse IgG instead of primary antibodies. Subsequently, the sections were successively incubated with biotinylated secondary antibodies and streptomyces anti-biotin protein-peroxidase at 25 °C for 10 min. The locations of the antigens were visualized with chromogen 0.1% diaminobenzidine tetrahydrochloride. Finally, all slices were counterstained with hematoxylin. All slices were prepared under the same conditions and time to ensure homogeneity between samples. The staining results were assessed with a Nikon Eclipse TE2000S microscope. Pictures were photographed at the identical threshold and gain settings, same light intensity, and same exposure time.

### Immunofluorescence double staining of tissue sections

Three samples at PN5 were included, and at least three slices with good morphology from each sample were selected for immunofluorescence analysis. The procedures before primary antibody incubation were the same as the methods of immunohistochemistry mentioned above. The slices were incubated overnight (15 h) with diluted KLF6 mouse monoclonal antibodies (1:50, Santa Cruz, # sc-7158) and then treated with FITC conjugated goat anti-mouse IgG (Boster, #BA1101). Subsequently, the slices were treated with goat serum, following which the slices were incubated overnight (15 h) with diluted P21 rabbit polyclonal antibody (1:100, Proteintech, # 28248-1-AP). Wash four times with PBS. The slices were treated with Cy3 conjugated goat anti-rabbit IgG (Boster, #BA1032) in an immunohistochemical wet box. All the slices were counterstained with DAPI dye (Beyotime, #C1006)) for 5 min away from light. A Leica SP8 confocal laser scanning microscope was used to observe the fluorescence intensity. All slices were prepared under the same conditions and time to ensure homogeneity between samples. Images were taken under identical gain and offset values, with the same intensity of excitation wavelength and exposure time.

### Cell culture

Professor Feng Wang provided the iMDP-3 cells. The cells were established, as stated previously.^28^ Primary dental papilla cells were obtained from molars of P3 C57BL/6 mice mandibles and subsequently transformed using Psv40. The resulting stable iMDP-3 cell line was often used in studying the mechanism of odontoblast differentiation.^21,40^ The cells were grown in Dulbecco’s modified Eagle’s medium (DMEM) (Hyclone) containing 10% fetal bovine serum at 37 °C and 5% carbon dioxide. To stimulate odontogenesis, DM with odontoblast inductive capacity was used (DMEM supplemented with 10% fetal bovine serum, 50 μg·mL^−1^ ascorbic acid, 10 mmol·L^−1^ sodium β-glycerophosphate, and 100 nmol·L^−1^ dexamethasone).

### Cell immunofluorescence and laser confocal microscopy analysis

For immunofluorescence double staining of KLF6 and P21, iMDP-3 cells were collected to create cell-climbing pieces. After being cultured in DM for 0 and 11 days, the pieces were promptly fixed and permeabilized. Subsequently, 3% bovine serum albumin was used to block the piece. Mouse monoclonal KLF6 (1:50) and rabbit polyclonal P21 antibodies (1:100) were used. After overnight incubation at 4 °C, the pieces were treated with secondary antibodies (Alexa Fluor Plus 488 goat anti-mouse and 594 goat anti-rabbit IgG, 1:300, Thermo Fisher Scientific, #A32723 and # A32740) for 60 min at 25 °C. To perform nuclear staining, the pieces were treated with DAPI reagent for 5 min at 25 °C. A Leica SP8 confocal laser scanning microscope was used to observe the fluorescence intensity. All sections were prepared under the same conditions and time to ensure homogeneity between samples. Images were taken under identical gain and offset values, with the same intensity of excitation wavelength and exposure time. Each experiment was performed triplicately.

In addition, to identify the odontogenic capability, iMDP-3 were grown in DM on cell-climbing pieces. The pieces were fixed and subsequently incubated with diluted ALP (1:500, Abcam, # ab95462), COL1 (1:500, Abcam, # ab6308), OPN (1:500, Abcam, # ab8448), OSX (1:500, Abcam, # ab209484), DMP1 (C-terminus) (1:200, Sigma-Aldrich, # MABD19) antibodies overnight, respectively. Subsequently, the pieces were treated with Alexa Fluor Plus 488 for 60 min. The next steps were similar to immunofluorescence double staining.

### ALP and ARS staining

ALP was stained using BCIP/NBT ALP staining kit (Beyotime), following the kit manual. For ARS staining, the cells were stained with a 2% ARS solution (Beyotime). The results were observed using a Nikon Eclipse TE2000S microscope. Each experiment was performed triplicately.

### Transient transfection assays

For overexpression or knockdown of *Klf6*, iMDP-3 were cultured in six-well plates (5 × 10^5^ cells per well). After cell attachment, the cells were transfected for 6 h with the *Klf6* expression vector (flag-hKLF6, Addgene) or its control vector (pCI-neo, Invitrogen) using Lipofectamine 3000 (Invitrogen) in Opti-MEM (Gibco) and following the manual. The cells were harvested for use in RT-qPCR, western blotting, MTT assay, flow cytometric analysis, and ChIP 48 h after transfection. Each experiment was performed triplicately.

### shRNA knockdown

To knock down *Klf6*, three different *Klf6* shRNAs and one control shRNA were obtained (Santa Cruz, #sc-38021). The shRNA with the most effective silencing effect targeting *Klf6* in the pilot experiment was chosen for subsequent experiments. iMDP-3 were cultured in DM for 3 days. The cells were then further transfected with *Klf6* or control shRNA using Lipofectamine 3000 in Opti-MEM and following the manual. The cells were harvested for use in RT-qPCR, western blotting, MTT assay, and flow cytometric analysis 24 h after transfection. Each experiment was performed triplicately.

### MTT assay

MTT Assay Kit (Abcam, #ab211091) was used. iMDP-3 were seeded into 96-well plates (2 × 10^3^ cells per well) and preincubated for 24 h. Moreover, 50 μL MTT reagent and serum-free medium were respectively added to each well. After 3 h of incubation, 150 μL MTT solvent was incubated with the plates for 15 min without light. The optical density was then measured at 590 nm. Each experiment was performed three times.

### Flow cytometric cell apoptosis and cell cycle analysis

The Annexin V-FITC/PI (4 A Biotech) assay was used to perform a cell apoptosis analysis with transfected iMDP-3 cells. iMDP-3 were collected and resuspended. Some of the suspended cells were stained with Annexin V-FITC and propidium iodide. The remaining cells were fixed with ethanol and subsequently stained with PI (Sigma-Aldrich) following the treatment with RNAaseA (BioFroxx) for 30 min at 37 °C. Analyses were performed using an Attune NxT Flow Cytometer (Thermo Fisher Scientific). Each experiment was performed triplicately.

### RT-qPCR

RNA was isolated from iMDP-3 cells with TRIzol (Invitrogen). SuperScript II reverse transcriptase (Invitrogen) was used to perform reverse transcription. RT-qPCR was conducted with SYBR Green (Thermo Fisher Scientific) on an ABI 7300 Real-Time PCR System (Applied Biosystems). Target gene levels were calculated using the 2^−^^∆∆Ct^ formula. The reference gene was β-actin. Primers are listed in Table. S1. Each experiment was performed triplicately.

### Western blotting

Cells were lysed using RIPA reagent (Santa Cruz). After 15 min of centrifugation at 13,000 r·min^−1^ to remove debris, BCA assay kits (Thermo Fisher Scientific) were used to determine the protein concentration. Equivalent proteins were added to a polyacrylamide gel electrophoresis system and transferred to polyvinylidene fluoride membranes. After blocking with the skimmed milk powder, the membranes were treated with primary antibodies. i.e., the KLF6 (1:800), P21 (1:800), or β-ACTIN (1:500, Santa Cruz, # sc-8432). The next day secondary antibodies (Bioss) were employed. ECL reagents (Thermo Fisher Scientific) and an enhanced chemiluminescence system (Bio-Rad) were used to visualize bands. The densities of bands were obtained, and relative protein levels were semi-quantified using ImageJ (National Institutes of Health, USA). Each experiment was performed triplicately.

### Dual-luciferase reporter assay

The sequence between nucleotides (nt) –739 and +100 (the *p21* promoter region) was subcloned into a pGL3-basic luciferase vector (Promega) to form a wild-type *p21* reporter plasmid. Each mutant *p21* plasmid was designed with a single deletion of the CCC/GGG at either nt −582, nt −79, nt +7, or nt +46. The recombinant plasmids were constructed by Genewiz (Suzhou, China), and the plasmid sequences were verified with DNA sequencing. iMDP-3 cells were co-transfected with pCI-neo; flag-hKLF6 plasmids; pGL3-basic, wild-type, or mutant *p21* reporter; or Renilla luciferase plasmid. After 24 h, the luciferase activities were detected using a dual-luciferase reporter assay (Promega) following the manual. Each experiment was performed triplicately.

### ChIP-qPCR

A ChIP kit (CST) was used to verify KLF6 binding sites. Histone-DNA cross-linking was induced by 37% formaldehyde. Cells were treated with lysis buffer containing protease inhibitors scraped and collected into tubes and then kept at 4 °C for 10 min. After ultrasonic fragmentation, the DNA was sheared to a length of 200–1 000 bp. Centrifugation was at 13 300 r·min^−1^, following which the supernatant was collected to new tubes. Moreover, 2% of the samples were set as inputs. Equivalent amounts of samples were diluted ten-fold by ChIP dilution buffer. Each sample was incubated with IgG antibody (1 μg·mL^−1^) at 4 °C (rotate slowly) to reduce background. After overnight incubation, 30 μL protein A agarose/salmon sperm DNA was added and incubated with samples for 60 min at 4 °C (rotate slowly) to pull down nonspecific binding complexes. The supernatants were carefully collected and subsequently incubated with KLF6 antibody overnight at 4 °C (rotate slowly). Subsequently, 30 μL protein A agarose/salmon sperm DNA was incubated with samples again to pull down immune complexes. Washing the beads consecutively with each buffer, including ice-cold lower salt, high salt, LiCl immune complex, and TE buffers. The histone complexes were eluted by elution buffer at 25 °C for 15 min. Histone-DNA crosslinks were reversed. Further, a core mix solution was added to each sample and the mixtures were incubated for 60 min at 45 °C. DNA purification was conducted by MicroElute DNA Clean-Up Kit (Omega), following the manual. Finally, quantitative PCR was performed. A pair of primers were synthesized to verify the *Klf6* binding sites on the p21 promoter: forward, 5′- GTCTGTTCAGTCCTGGGTG-3′; reverse, 5′-TTCTGCTGGCAAAGTGGG-3′. Each experiment was performed triplicately.

### Construction of rat pulp exposure models

Eighteen male Wistar rats (9 weeks old, 200–250 g each) were purchased from Zhejiang University. The rats were injected with pentobarbital sodium (100 mg·kg^−1^, intraperitoneal) and randomly divided into three groups: (1) the pulpitis group; (2) the direct capping group; and (3) the negative control group. For the pulpitis group, the maxillary first molars were drilled open on the occlusal face using a no. 1/4 round bur under water cooling. For the direct capping group, the maxillary first molars were drilled open in the same way and pulp capping material (mineral trioxide aggregate, MTA, Dentsply Endodontics) was placed into the perforations. Subsequently, all the cavities were filled with glass ionomer cement (Fuji IX). The control group only received the anesthesia (same procedure) without any other procedures. The rats were killed 4weeks after pulp exposure. The maxillae were harvested for immediate fixation and then decalcified for 8 weeks. Histological sections were prepared. Consecutive serial sections with the representative morphologies of each group were processed with H&E, immunohistochemical, or immunofluorescence double staining. The procedures of these staining methods and image capture followed the same steps described above.

### Statistical analyses

All data are presented as the mean ± standard deviation and analyzed using SPSS (version 26.0, software, IBM). Levene’s test was performed first to analyze the variance homogeneity. An independent samples *t*-test was used to determine the difference between the two groups. All the significant differences were determined as follows: *P < 0.05, ** *P* < 0.01, and *** *P* < 0.001.

## Supplementary information

Table S1

Table S2

Figure S1

Figure S2

Figure S3

Figure legends of supplemental figures

## Data Availability

All data associated with this study are presented in the paper.

## References

[1] Caton J, Tucker AS (2009). Current knowledge of tooth development: patterning and mineralization of the murine dentition. J. Anat..

[2] Kovacs CS (2021). The role of biomineralization in disorders of skeletal development and tooth formation. Nat. Rev. Endocrinol..

[3] Chen Y, Zhang Y, Ramachandran A, George A (2016). DSPP is essential for normal development of the dental-craniofacial complex. J. Dent. Res..

[4] Chmilewsky F (2019). C5L2 regulates DMP1 expression during odontoblastic differentiation. J. Dent. Res..

[5] Balic A, Thesleff I (2015). Tissue interactions regulating tooth development and renewal. Curr. Top. Dev. Biol..

[6] Presnell JS, Schnitzler CE, Browne WE (2015). KLF/SP transcription factor family evolution: expansion, diversification, and innovation in eukaryotes. Genome Biol. Evol..

[7] Chen Z (2009). Spatial and temporal expression of KLF4 and KLF5 during murine tooth development. Arch. Oral. Biol..

[8] Chen Z (2016). Klf10 regulates odontoblast differentiation and mineralization via promoting expression of dentin matrix protein 1 and dentin sialophosphoprotein genes. Cell. Tissue Res..

[9] Chen Z (2017). Klf5 mediates odontoblastic differentiation through regulating dentin-specific extracellular matrix gene expression during mouse tooth development. Sci. Rep..

[10] Han N, Chen Z, Zhang Q (2016). Expression of KLF5 in odontoblastic differentiation of dental pulp cells during in vitro odontoblastic induction and in vivo dental repair. Int. Endod. J..

[11] Lin H (2013). KLF4 promoted odontoblastic differentiation of mouse dental papilla cells via regulation of DMP1. J. Cell. Physiol..

[12] Tao H (2019). Klf4 Promotes dentinogenesis and odontoblastic differentiation via modulation of TGF-β signaling pathway and interaction with histone acetylation. J. Bone Miner. Res..

[13] Koritschoner NP (1997). A novel human zinc finger protein that interacts with the core promoter element of a TATA box-less gene. J. Bio. Chem..

[14] DiFeo A, Martignetti JA, Narla G (2009). The role of KLF6 and its splice variants in cancer therapy. Drug Resist. Updat..

[15] Narla G (2007). In vivo regulation of p21 by the Kruppel-like factor 6 tumor-suppressor gene in mouse liver and human hepatocellular carcinoma. Oncogene.

[16] Andreoli V, Gehrau RC, Bocco JL (2010). Biology of Kruppel-like factor 6 transcriptional regulator in cell life and death. IUBMB Life..

[17] Wu Z, Wang S (2013). Role of kruppel-like transcription factors in adipogenesis. Dev. Biol..

[18] Lu XJ, Shi Y, Chen JL, Ma S (2015). Kruppel-like factors in hepatocellular carcinoma. Tumour Biol..

[19] Syafruddin SE (2019). A KLF6-driven transcriptional network links lipid homeostasis and tumour growth in renal carcinoma. Nat. Commun..

[20] Mgbemena V (2012). Transactivation of inducible nitric oxide synthase gene by Kruppel-like factor 6 regulates apoptosis during influenza A virus infection. J. Immunol..

[21] Mgbemena V, Segovia J, Chang TH, Bose S (2013). KLF6 and iNOS regulates apoptosis during respiratory syncytial virus infection. Cell Immunol..

[22] Narla G (2001). KLF6, a candidate tumor suppressor gene mutated in prostate cancer. Science.

[23] Zhang X (2012). MicroRNA-181a promotes gastric cancer by negatively regulating tumor suppressor KLF6. Tumour Biol..

[24] Rane MJ, Zhao Y, Cai L (2019). Krupsilonppel-like factors (KLFs) in renal physiology and disease. EBioMedicine.

[25] Pu FF (2021). SP1-induced long non-coding RNA SNHG6 facilitates the carcinogenesis of chondrosarcoma through inhibiting KLF6 by recruiting EZH2. Cell Death. Dis..

[26] Matsumoto N (2006). Developmental regulation of yolk sac hematopoiesis by Kruppel-like factor 6. Blood.

[27] Chen Z, Xie H, Yuan J, Lan Y, Xie Z (2021). Krüppel‐like factor 6 promotes odontoblastic differentiation through regulating the expression of dentine sialophosphoprotein and dentine matrix protein 1 genes. Int. Endo. J..

[28] Wang F (2013). Immortalized mouse dental papilla mesenchymal cells preserve odontoblastic phenotype and respond to bone morphogenetic protein 2. Vitr. Cell Dev. Biol. Anim..

[29] Yu T, Klein OD (2020). Molecular and cellular mechanisms of tooth development, homeostasis and repair. Development.

[30] Ishikawa Y, Ida-Yonemochi H, Nakakura-Ohshima K, Ohshima H (2012). The relationship between cell proliferation and differentiation and mapping of putative dental pulp stem/progenitor cells during mouse molar development by chasing BrdU-labeling. Cell. Tissue Res..

[31] Iwamoto T (2017). Pannexin 3 regulates proliferation and differentiation of odontoblasts via its hemichannel activities. PLoS ONE.

[32] Bloch‐Zupan A, Leveillard T, Gorry P, Fausser J, Ruch J (1998). Expression of p21^WAF1/CIP1^ during mouse odontogenesis. Eur. J. Oral. Sci..

[33] Jernvall J, Aberg T, Kettunen P, Keranen S, Thesleff I (1998). The life history of an embryonic signaling center: BMP-4 induces p21 and is associated with apoptosis in the mouse tooth enamel knot. Development.

[34] Nakatomi M, Ida-Yonemochi H, Ohshima H (2013). Lymphoid enhancer-binding factor 1 expression precedes dentin sialophosphoprotein expression during rat odontoblast differentiation and regeneration. J. Endod..

[35] Zhao Y (2021). lncRNA-Xist/miR-101-3p/KLF6/C/EBPα axis promotes TAM polarization to regulate cancer cell proliferation and migration. Mol. Ther. Nucleic Acids.

[36] Liang WC (2014). Identification of miRNAs that specifically target tumor suppressive KLF6-FL rather than oncogenic KLF6-SV1 isoform. RNA Biol..

[37] Su Y (2011). Inhibition of proliferation of rat lens epithelial cell by overexpession of KLF6. Mol. Vis..

[38] Singh SK, Banerjee S, Acosta EP, Lillard JW, Singh R (2017). Resveratrol induces cell cycle arrest and apoptosis with docetaxel in prostate cancer cells via a p53/p21^WAF1/CIP1^ and p27^KIP1^ pathway. Oncotarget.

[39] Li K (2021). Cyclin-dependent kinases-based synthetic lethality: Evidence, concept, and strategy. Acta Pharm. Sin. B..

[40] Alao JP (2007). The regulation of cyclin D1 degradation: roles in cancer development and the potential for therapeutic invention. Mol. Cancer.

[41] Benzeno S (2004). Cyclin-dependent kinase inhibition by the KLF6 tumor suppressor protein through interaction with cyclin D1. Cancer Res..

[42] Caiaffa KS (2019). Effect of analogues of cationic peptides on dentin mineralization markers in odontoblast-like cells. Arch. Oral. Biol..

[43] Lang U (2013). GSK3β phosphorylation of the KLF6 tumor suppressor promotes its transactivation of p21. Oncogene.

[44] Li D (2005). Regulation of Krüppel-like factor 6 tumor suppressor activity by acetylation. Cancer Res..

[45] Xia K (2020). RGD-and VEGF-mimetic peptide epitope-functionalized self-assembling peptide hydrogels promote dentin-pulp complex regeneration. Int. J. Nanomed..

[46] Hui T (2018). EZH2 regulates dental pulp inflammation by direct effect on inflammatory factors. Arch. Oral. Biol..

