# Correction To: KLF6 facilitates differentiation of odontoblasts through modulating the expression of P21 in vitro

**DOI:** 10.1038/s41368-022-00201-4

**Published:** 2022-09-21

**Authors:** Zhuo Chen, Wenzhi Wu, Chen Zheng, Yanhua Lan, Huizhi Xie, Zhijian Xie

**Affiliations:** Stomatology Hospital, School of Stomatology, Zhejiang University School of Medicine, Clinical Research Center for Oral Diseases of Zhejiang Province, Key Laboratory of Oral Biomedical Research of Zhejiang Province, Cancer Center of Zhejiang University, Hangzhou, China

**Keywords:** Differentiation, Cell proliferation

Correction to: *International Journal of Oral Science* 10.1038/s41368-022-00172-6, published online 14 April 2022

**Following publication of this article, the authors reported the below errors**.

The third section of RESULTS: Effects of Klf6 overexpression on gene expression, proliferation, apoptosis, and cell cycle.

Paragraph 4: The *Klf6* overexpression group had a lower average proportion of G0/G1 phase cells (52.77% ± 2.25%) (*P* < 0.05). In the control group, that proportion was 62.07% ± 2.95%. Furthermore, 35.62% ± 1.95% and 25.91% ± 1.17% of the cells in the *Klf6* overexpression and control groups were in the S phase, respectively (*P* < 0.05). The G2/M phase proportions were 11.63% ± 0.30% and 12.53% ± 1.29% for the *Klf6* overexpression and control groups, respectively (*P* > 0.05).

Corrected to: The control group had a lower average proportion of G0/G1 phase cells (52.77% ± 2.25%) (*P* < 0.05). In the *Klf6* overexpression group, that proportion was 62.07% ± 2.95%. Furthermore, 35.62% ± 1.95% and 25.91% ± 1.17% of the cells in the control and *Klf6* overexpression groups were in the S phase, respectively (*P* < 0.05). The G2/M phase proportions were 11.63% ± 0.30% and 12.53% ± 1.29% for the control and *Klf6* overexpression groups, respectively (*P* > 0.05).

The original article was updated.

