# Supplementary material for: KLF6 facilitates differentiation of odontoblasts through modulating the expression of P21 in vitro
Source: Int J Oral Sci. 2022 Apr 14;14:20. doi: 10.1038/s41368-022-00172-6 (PMC9010434; doi:10.1038/s41368-022-00172-6)
Supplement: Supplementary file 1 — Table S1 [file 41368_2022_172_MOESM1_ESM.docx]

| Genes | Forward | Reverse | Size (bp) |
| --- | --- | --- | --- |
| *Klf6* | 5’-CCACTTGAAAGCACATCAGCG-3’ | 5’-TTCGGAAGTGTCTGGTCAACTC-3’ | 113 |
| *p21* | 5’- TAAGGACGTCCCACTTTGCC-3’ | 5’-CGTCTCCGTGACGAAGTCAA-3 | 197 |
| *p27* | 5’-AGATACGAGTGGCAGGAGGT-3’ | 5’-ATGCCGGTCCTCAGAGTTTG-3’ | 171 |
| *Ccnd1* | 5’-TCAAGTGTGACCCGGACTG-3’ | 5’-ATGTCCACATCTCGCACGTC-3’ | 175 |
| *Dspp* | 5’-AACTCTGTGGCTGTGCCTCT-3’ | 5’-AACTCTGTGGCTGTGCCTCT-3’ | 171 |
| *Dmp1* | 5’-CAGTGAGGATGAGGCAGACA-3’ | 5’-TCGATCGCTCCTGGTACTCT-3’ | 175 |

Table S1. Primer sequences used in this study

bp, base pairs.
