# Supplementary material for: KLF6 facilitates differentiation of odontoblasts through modulating the expression of P21 in vitro
Source: Int J Oral Sci. 2022 Apr 14;14:20. doi: 10.1038/s41368-022-00172-6 (PMC9010434; doi:10.1038/s41368-022-00172-6)
Supplement: Supplementary file 2 — Table S2 [file 41368_2022_172_MOESM2_ESM.docx]

Table S2. Sequences of potential *Klf6*-binding sites on *p21* promoter.

| Initiation sites | Sequences |
| --- | --- |
| nt -582 | Forward:5’-TTTCCTGTGGGTGATGGGAT-3’ |
| nt -79 | Forward:5’-TCAGTCCTGGGTGGGGACTA-3’ |
| nt +7 | Forward:5’-TCAGTGCAGGGTGGTGGAGA-3’ |
| nt +46 | Forward:5’-CAGCTGTGGGGTGAGGAGGA-3’ |

nt, nucleotides
