# Supplementary figures and images for: KLF6 facilitates differentiation of odontoblasts through modulating the expression of P21 in vitro
Source: Int J Oral Sci. 2022 Apr 14;14:20. doi: 10.1038/s41368-022-00172-6 (PMC9010434; doi:10.1038/s41368-022-00172-6)

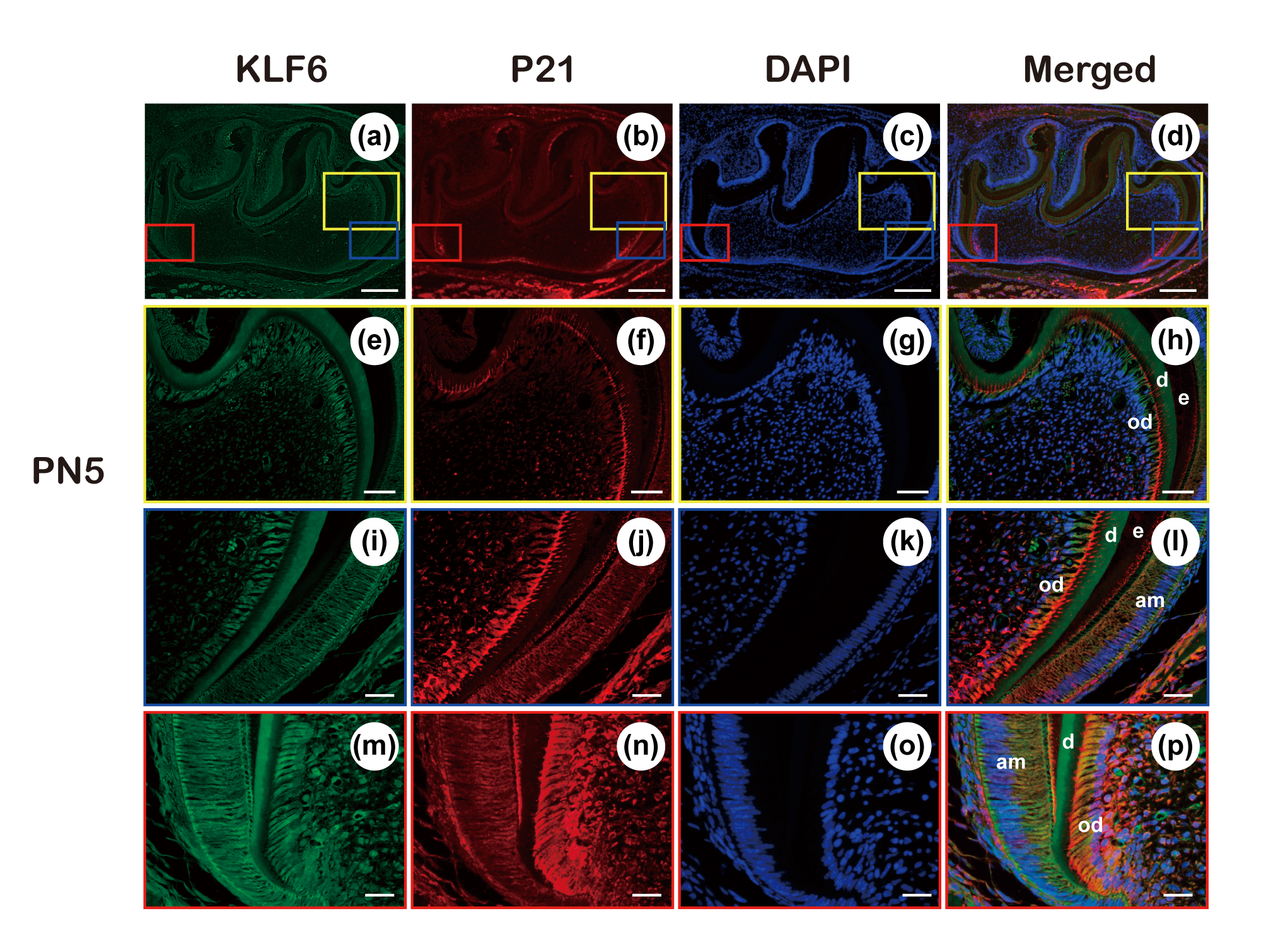

Supplement: Supplementary file 3 — Figure S1 [file 41368_2022_172_MOESM3_ESM.tif]

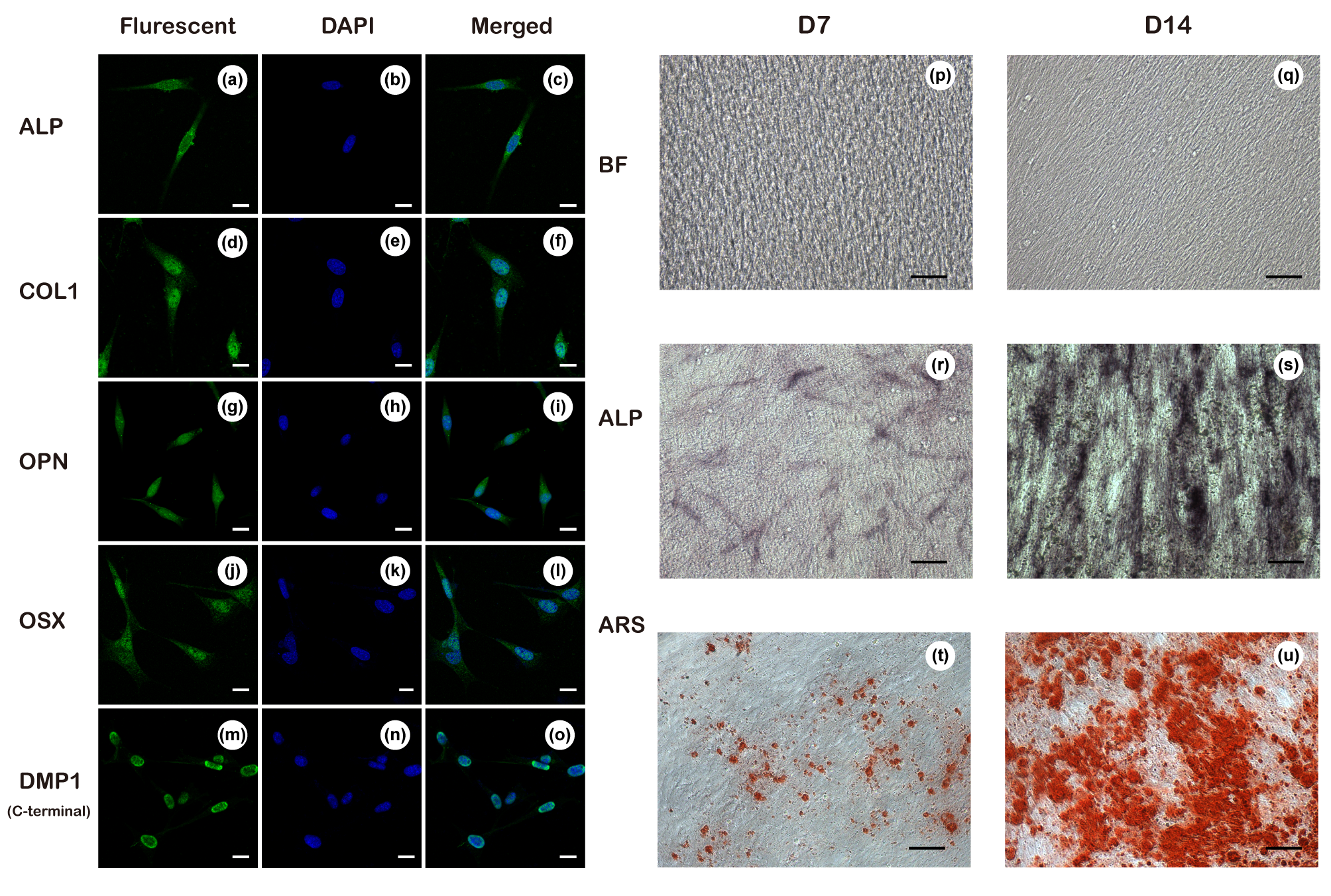

Supplement: Supplementary file 4 — Figure S2 [file 41368_2022_172_MOESM4_ESM.tif]

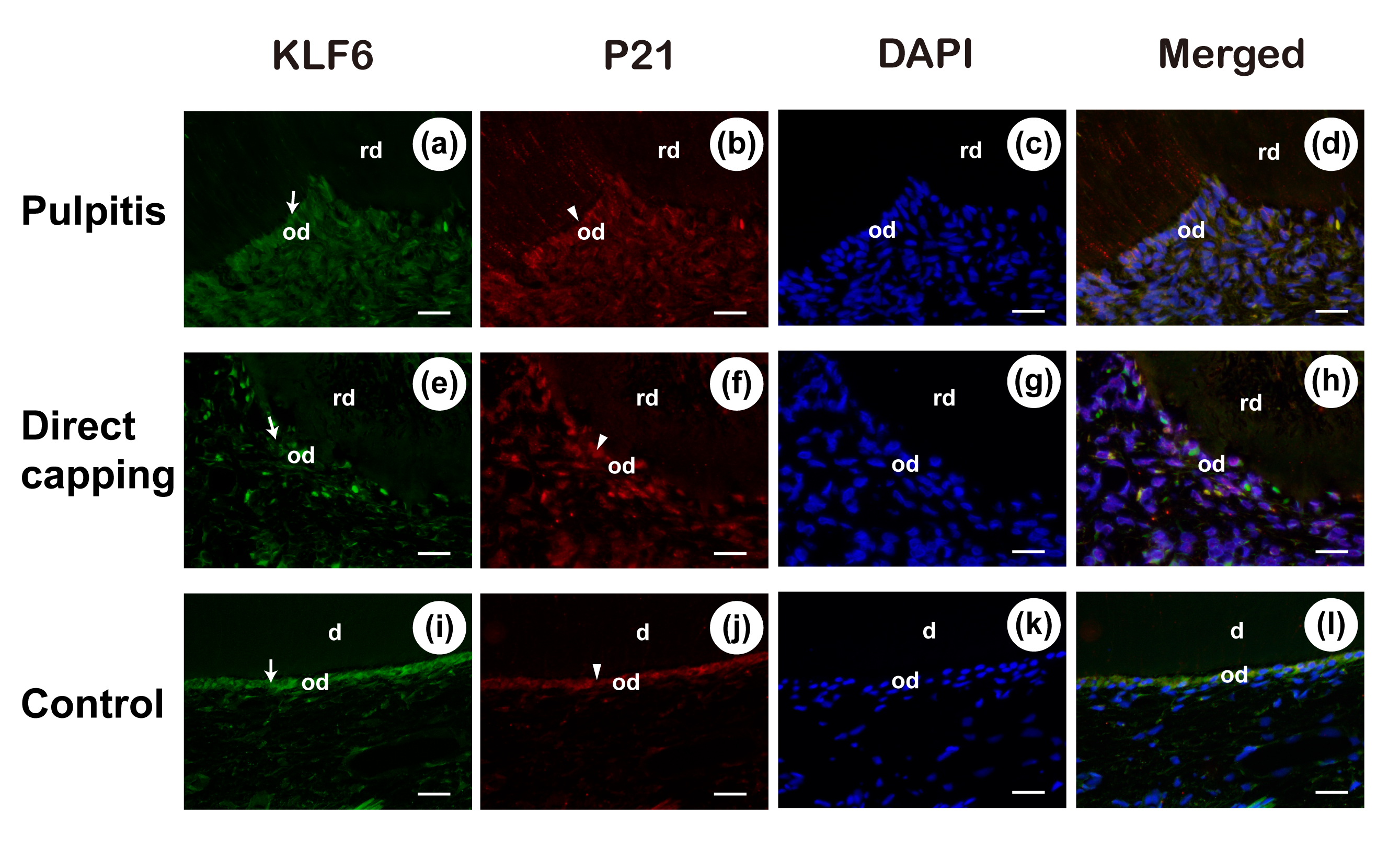

Supplement: Supplementary file 5 — Figure S3 [file 41368_2022_172_MOESM5_ESM.tif]
