# Supplementary material for: KLF6 facilitates differentiation of odontoblasts through modulating the expression of P21 in vitro
Source: Int J Oral Sci. 2022 Apr 14;14:20. doi: 10.1038/s41368-022-00172-6 (PMC9010434; doi:10.1038/s41368-022-00172-6)
Supplement: Supplementary file 6 — Figure legends of supplemental figures [file 41368_2022_172_MOESM6_ESM.docx]

**Supplementary Figure Legends**

**Figure S1. Immunofluorescence staining of KLF6 and P21 in mouse dental papilla mesenchymal cells at PN5 in vivo. (a-d)** Immunofluorescence staining of KLF6 and P21 in murine tooth germs at PN5. **(e-h)** Higher magnification images of yellow-boxed areas in (a-d), respectively. **(i-l)** Higher magnification images of blue-boxed areas in (a-d), respectively. **(m-p)** Higher magnification images of red-boxed areas in (a-d), respectively. e, enamel; d, dentine; am, ameloblast; od, odontoblast. Bars = 200 μM (a-d), 50 μM (e-h), 20 μM (i-p).

**Figure S2.** **iMDP-3 cells maintain the ability of differentiating into odontoblasts and secreting mineralized matrix.** Immunofluorescence staining of ALP **(a)**, COL1 **(d)**, OPN **(g)**, OSX **(j)** and DMP1 **(m)** in iMDP-3 cells after culturing the cells in DM for 14 days. **(b, e, h, k, n)** Nuclei of iMDPC-3 cells with DAPI. **(c, f, i, l, o)** Merged images. **(p, q)** Light microscopy micrographs of cells. ALP staining at day 7 **(r)** and day 14 **(s)**. Alizarin Red staining at day 7 **(t)** and day 14 **(u)**. BF, bright filed. Bars = 20 μM.

**Figure S3. Immunofluorescence double staining of KLF6 and P21 in the rat pulp exposure model.** KLF6 **(a, e)** and P21 **(b, f)** were positive in odontoblasts and dental pulp cells around reparative dentine in the dental pulpitis group and direct capping group. In the control group, KLF6 **(i)** and P21 **(j)** were mainly expressed in odontoblasts. **(c, g, k)** DAPI staining. **(d, h, l)** Merged images. Arrows, positive KLF6 expression in odontoblasts; Arrowheads, positive P21 expression in odontoblasts. d, dentine; rd, reparative dentine; od, odontoblast. Bars = 20 μM.
